# On Mechanical Power in the Cure of Hernia, Practically Adapted to Professional Students, and Those Afflicted with Ruptures

**Published:** 1831

**Authors:** 


XLII.

ON MECHANICAL POWER IN THE CURE OF HERNIA, PRACTICALLY ADAPTED TO PROFESSIONAL STUDENTS, AND THOSE AFFLICTED WITH RUPTURES. By Jos. Egg, &c. 12mo, pp. 38. 1831.

WE happened lately to be interested in the application of a truss to a recent hernia in a young person, and took occasion to examine the principles on which Mr. Egg's trusses are made and employed. It is hardly necessary to remind surgeons that the general principle on which trusses are constructed, is *pressure* to keep the intestine within the parietes of the abdomen, *and* to prevent its protrusion when any muscular exertion is used. Now we confess our conviction that the *pressure* is unnecessary, if not prejudicial; and that the grand salutary operation required is *resistance*—a very different thing from pressure. Where the pressure is produced, as it usually is, by a very elastic spring, it is obvious that *resistance* to protrusion is limited, and may be overcome. But suppose there was no elasticity whatever in the truss —that, for instance, an iron poker was bent round the body and accurately fitted to its circumference, so as to just touch the aperture through which the hernia descends, then there would be *resistance* to protrusion, and complete security, without any, or with very little pressure. This is the principle on which Mr. Egg fabricates and adapts his trusses, and from what we have seen, we believe he is correct and consequently successful. We shall introduce an extract or two from the little pamphlet before us.

"The principle of all trusses, which are constructed for this purpose, is pressure; and this pressure it is endeavoured to increase, or to diminish, not according to the supposed necessity of the patient, but according to what the patient may be able to bear; and, as the physical power to endure rarely equals the necessity for endurance, the mere Truss-maker has no means of adapting the power to the necessity, relief rarely, and a cure still more rarely, can be hoped for. Indeed, when a cure has, by this application, been performed, it is wholly the result of accident, without any reference to the skill either of the fabricator of the truss, or of the person who applies it to the part which requires such assistance. In point of fact, a bandage, on the common principle, has the same chance of efficacy as a truss. It may happen to afford the desired support and pressure, on the exact point where it is required; and in such an event, not only relief, but even permanent cure will be the result, unless the extreme age of the patient have deprived nature of its restorative power."

"Herniæ, or Ruptures, demand powers as varying as themselves; but it generally happens that, in proportion to the greater pressure required to effect the cure is the extreme sensitiveness of the morbid part; so that, as its call for mechanical pressure increases, its means of endurance diminish: hence the state of the sufferer was utterly hopeless, until the merest accident disclosed the means of affording permanent relief and cure to their present possessor."

"Suppose the extreme case of a person afflicted with Hernia, which requires a pressure equal to the weight of fifteen pounds; when the infirmity of the part will disable the sufferer from existing, under an application which shall exceed three pounds,—what chance of relief can he, reasonably, have? Besides, every truss, in common use, acts by pressure; therefore, to annihilate the elasticity of that spring, on the pressure of which depends the sole chance of relief, is to convert it into a mere bandage, and that bandage utterly inadequate to effect what only it professes to accomplish.

The common truss, particularly that which is designated 'Mechanical,' and acts solely by pressure, is so constructed that, if its pressure equal a weight of seven pounds on the ruptured part, it presses in an equal degree upon the spine, where pressure is not only not wanted, but cannot be sustained, without the risk of very serious danger. I have been applied to by persons whose spines have been brought into an unequivocal state of decay, from the constant pressure inflicted, by this 'Mechanical' truss, upon the part.

Of a somewhat better description, is the truss which, being strapped round the body, is reduced to a mere bandage; for although this may compress the shape, so as to give it a wasp-like appearance, it still distributes the pressure, throughout the entire round, with some degree of equality, and is not so likely to become injurious, though its efficacy, even as to relief (much less cure), may very well be questioned; neither is it always freed from the infliction of even severe injuries.

Another truss is designated the 'Spring Truss.' This is likewise strapped on, as tight as the spring will permit; of course, by this method of fastening, it becomes a bandage, and, until it is fastened, the spring does not act at all. In short, the trusses, one and all (excepting only that which I have introduced, and which is solely manufactured by, and to be obtained from myself) act entirely by pressure, which they have no means of regulating, other than by tightening or relaxing the straps. Beyond this, they, it is true, can change the spring for one of greater power, or supply an additional spring; but all these contrivances only serve to increase the torture of their already agonised patients, without giving them any facilities of endurance; and we still are forced to recur to Sir Astley Cooper's anxious research—for a strong truss, and an easy one!"

---

XLIII.

